# Risk factors for complications in peripheral intravenous catheters in adults: secondary analysis of a randomized controlled trial**^1^**

**DOI:** 10.1590/1518-8345.1457.2833

**Published:** 2016-11-28

**Authors:** Derdried Athanasio Johann, Mitzy Tannia Reichembach Danski, Stela Adami Vayego, Dulce Aparecida Barbosa, Jolline Lind

**Affiliations:** 2 PhD, RN, Instituto Federal do Paraná, Curitiba, PR, Brazil.; 3 PhD, Adjunct Professor, Departamento de Enfermagem, Universidade Federal do Paraná, Curitiba, PR, Brazil.; 4 PhD, Adjunct Professor, Departamento de Estatística, Universidade Federal do Paraná, Curitiba, PR, Brazil.; 5 PhD, Associate Professor, Escola Paulista de Enfermagem, Universidade Federal de São Paulo, São Paulo, SP, Brazil.; 6 Master's student, Programa de Pós-Graduação em Enfermagem, Universidade Federal do Paraná, Curitiba, PR, Brazil.

**Keywords:** Catheterization, Peripheral, Risk Factors, Complications, Randomized Controlled Trial, Nursing

## Abstract

**Objective:**

analyze the risk factors linked to complications in peripheral intravenous catheters.

**Method:**

secondary data analysis of a randomized controlled trial with 169 medical and surgical patients placed in two groups, one with integrated safety catheter (n=90) and other using simple needle catheter (n=79), with three months follow-up time.

**Results:**

the risk factors that raised the odds of developing complications were: hospitalization between 10-19 days (p=0.0483) and 20-29 days (p=0,0098), antimicrobial use (p=0.0288) and use of fluid solutions (p=0.0362). The 20 Gauge lowered the risks of complications (p=0.0153). Multiple analysis showed reduction of risk for the 20 Gauge (p=0.0350); heightened risk for solutions and fluids (p=0.0351) and use of corticosteroids (p=0.0214).

**Conclusion:**

risk factors linked to complications in peripheral intravenous catheters were: hospitalization periods between 10-29 days, antimicrobial infusion, solutions and fluids and corticosteroids. Regarding complications, 20 Gauge is a protecting factor compared with 22. Brazilian Clinical Trials Registry: RBR-46ZQR8.

## Introduction

Intravenous therapy is commonly used in hospitals, by inserting peripheral intravenous catheters. Most catheters are removed due to the occurrence of complications, end of treatment or absence of use^1^. The estimated annual use is about 200 million catheters in the United States of America (USA)^2^. In Spain, approximately half of hospitalized patients receive an intravenous catheter, being 95% of them peripheral^3^. More than 70% of patients admitted to hospitals require peripheral intravenous catheters^2^. Other studies show the use of peripheral venous catheters in 86.4%^4^ and 80.6%^5^ of the patients.

In spite of this extended use, the use of peripheral venous catheters can lead to complications such as phlebitis, obstruction, seepage, leakage and accidental removal^6^, resulting in increased hospitalization and treatment costs, and patient discomfort^1^. Understanding the risk factors for developing complications can facilitate the task of daily care of the nursing team, and may help to produce knowledge and scientific evidence to support the decision making of the nurses geared towards minimizing the risk of peripheral intravenous therapy. Thus, the general purpose of this secondary analysis was to analyze the risk factors related to the occurrence of complications in the peripheral venous catheterization and the specific objective was to compare the incidence of complications according to the type of peripheral venous catheter used: integrated safety catheter and simple needle catheter. The main study, from which this secondary analysis was derived, consisted in a randomized clinical trial that analyzed the complications arising from the use and type of peripheral venous catheters in adults^7^ ^-^ ^8^.

## Methods

This is a secondary analysis of a randomized clinical trial, in which randomization occurred by systematic random sampling, in two groups: integrated safety catheter and simple needle catheter. The integrated safety catheter consists of a silicon covered needle with double angle, tri-faceted bezel connected to the mandrel through metal guide and handle; made of polyurethane biomaterial; it has full protection of the needle device, activated after the puncture; wings with slots; transparent vinyl extender tube; bio-selective reflux chamber filter cover; fast cutting clamp; two-way access composed of female "Y" connector, one a Luer-Lok^(r)^ connection and another with removable male plug device. The short flexible catheter is of the needle type, with internal safety device (triggered passively) and single-use, disposable flip, needing an extender attached to it to let the infusion occur; the extenders used in the research institution had valves, simple equipment and intermediate extenders, and two/four ways access. The local complication of peripheral venous catheterization variable was the primary outcome and included the occurrence of phlebitis, thrombophlebitis, extravasation, seepage, obstruction, accidental traction and catheter site infection, evaluated according to international guidelines^6^. The risk factors inherent to the development of local complications in peripheral venous catheterization were secondary outcomes.

The survey was conducted in medical and surgical units of a large university hospital in Curitiba-PR, Brazil. Participants were adult patients over eighteen years of age, needing peripheral intravenous therapy. The objects of the study were peripheral venous catheters, gauges 20 and 22 (G). Criteria for inclusion of participants: patients needing peripheral venous access for IV therapy; hospital stay forecasted to be more than 96 hours for medical and/or surgical treatment; one only inclusion in the study. Exclusion criteria were: peripheral venipuncture impeded by the presence of capillary fragility, and clinical conditions or local alterations that impaired peripheral venipuncture. It is noteworthy to remark that the participation in the research was subject to authorization of subjects or first degree relative by signing the Informed Consent Form.

Collectors were trained prior to data collection, a team of two PhD students, two masters graduates, four academic nursing and two nurses employees. They were trained through meetings, lasting between one and two hours each, in order to standardize the collection data and concepts addressed in this study (a 30 hours overall duration), as well as during the pilot test run, which was carried out in pairs (a collector and a researcher). The nursing staff of the units under survey also participated in this training. There were 34 meetings, lasting 40 to 60 minutes, with attendance of of 109 employees, and it occurred by dialogued lecture (standardized concepts according to international guidelines)^6^, illustrative video viewing and venipuncture workshop.

The collection period was from August to November 2014, reaching the number of participants proposed by the sample calculation. To calculate the sample it was considered an estimated prevalence of local complications related to peripheral catheter in 52% for the integrated safety catheter group, based on pilot study data. The sample size, an estimated 150 patients (75 in each group) ensured a 0.80 power (1 - β = 0.80) for detecting a minimum difference of 20% between treatments at the 0.05 significance level (α = 0, 05). The daily collection was done in pairs. In the collection there was replacement of materials, update of the list of inpatients and authorization request (informed consent), analysis of inclusions and randomization, reading of records, active search for participants, direct observation of the punctured catheter in the patient, and control of complications. A separate structured form collected data containing socio-demographic, clinical, and catheter-related variables; as well as outcomes. The patient was followed every day from the inclusion in research to the catheter removal.

In the analysis of descriptive data we used absolute and percentage frequencies and measures of central tendency and dispersion (average and standard deviation). In univariate analysis, the characteristics of the catheter groups were compared using the chi-square test, Fisher, Williams G, Mann-Whitney U and the binomial test of proportions. In all tests it was established a significance level of 5%. It was applied to calculate the relative risk (RR) and confidence interval (CI), jointly to multiple analysis to estimate the degree of association between variables. The reference category was indicated in the tables of results using the value 1 for the values of RR. The multivariate analysis was performed with the variables with p < 0.20 and were analyzed together to obtain a final model, using the Poisson regression model. The two groups were compared according to the following variables: (a) socio-demographic: identification, registration, gender, age, ethnicity, education, occupation and inpatient unit; (b) clinical: length of stay, clinical diagnosis, comorbidities, type of surgery (if present), presence of concomitant infection and its location, family history of disease, smoking and alcohol use and outcome of hospitalization - discharge/transfer or death; (c) data related to the catheter: date, time and number of puncture attempts, length of time in days, anatomical site of catheter insertion, catheter use, reasons for withdrawal, events, daily exchange and type of fixation used; and (d) outcome: complications - phlebitis (including grade), thrombophlebitis, extravasation, seepage, accidental traction, obstruction and local infection. The development of the study met national and international standards of ethics in research involving human subjects and obtained the Brazilian Clinical Trials Registry number RBR-46ZQR8.

## Results

The study include 193 eligible participants, from them 15 were excluded from the data analysis (18 G gauge, puncture in different location of the upper limbs, exudation in limbs and outlier - length of stay longer than 400 days - making it impossible to compare the groups); nine participants refused or gave up participation (eight of them after randomization), and 169 patients were included (90 in the group with integrated security catheter and 79 in the group with single needle catheter); It evaluated only one catheter per patient; and records were suppressed if they were not informed of the statistical analysis. In the two analyzed catheter groups, the sample was homogeneous and characterized mostly by randomized ethnicity, approximate age of 50 years old, non-smoker and non drinker.

In the sample, the predominance fell in the males' medicine clinical ward, clinical diagnosis of digestive diseases, no comorbidity associated, non-surgical procedures during hospitalization, absence of infectious process and discharge as outcome. Regarding length of stay, there was a longer duration (in days) in the group with integrated safety catheter (Table 1). The higher prevalence in both groups was of catheters caliber 20; location in upper left arm, forearm region and puncture success in the first attempt. With respect to the purpose of those devices, the prevalence fell on infusion of solutions and fluids, sedatives and analgesics and other drugs (Table 1), but a minority of antibiotics, electrolytes, anticoagulants, vesicant drugs and corticosteroids were also used. Regarding the time of use, the majority of inserted catheters remained a time equal to or exceeding 72 hours (Table 1). Among the reasons for withdrawal, hospital discharge, followed by phlebitis were predominant. (Table1).

**Table 1:**
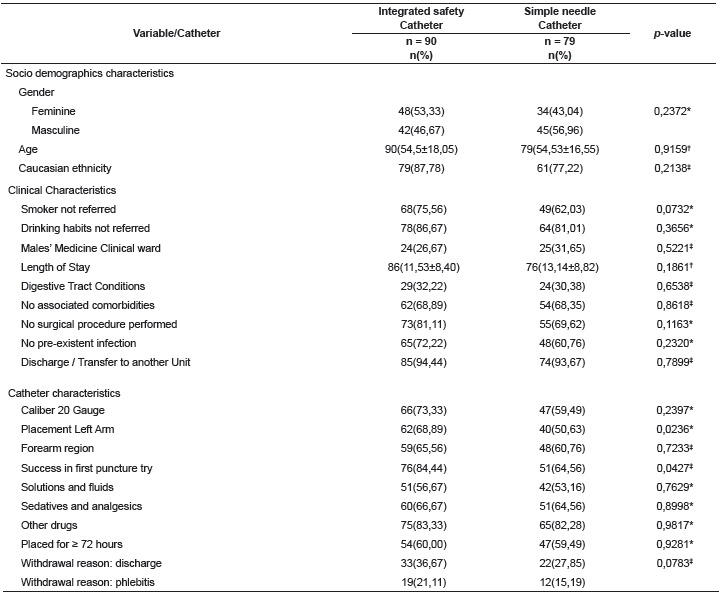
Characteristics: socio demographic, clinical and linked to catheter groups. Curitiba, PR, Brazil, 2014

* Chi- Square Test ^†^ Mann-Whitney U Test; ^‡^ Williams G Test

Complications rates are shown in Table 2. No statistically significant difference was found between the two groups related to complications. It is noteworthy that six cases of local infection occurred, one of extravasation and one thrombophlebitis, which were left out of univariate analysis as they represented a small number in the sample.

**Table 2:**
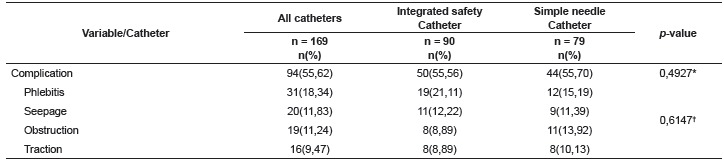
Distribution of complications in both groups. Curitiba, PR, Brazil, 2014

* Binomial proportion Test; ^†^ Williams G Test

The onset of complications during intravenous therapy may be attributed to several factors. In analyzing the risk factors, among clinical variables, the length of stay from 10 to 19 days increases the risk of developing complications in 1.36 (p = 0.0483) and when this length is extended between 20 to 29 days, the risk increases in 1.61 (p = 0.0098) compared to the period from 1 to 9 days. There were no risk factors associated with socio-demographic characteristics. Related to catheter variables, gauge 20 reduces the risk of complications in 0.71 (p = 0.0153) compared to gauge 22; the use of antimicrobials increases risk by 1.33 (p = 0.0288), in the same way that the infusion of solutions and fluids increases it by 1.32 (p = 0.0362) (Table 3). The risk factors of the most frequent complications (phlebitis, seepage, obstruction and traction) were analyzed by comparing the variables related to the data of catheters among those removed without complications and those with other complications (Table 3).

**Table 3:**
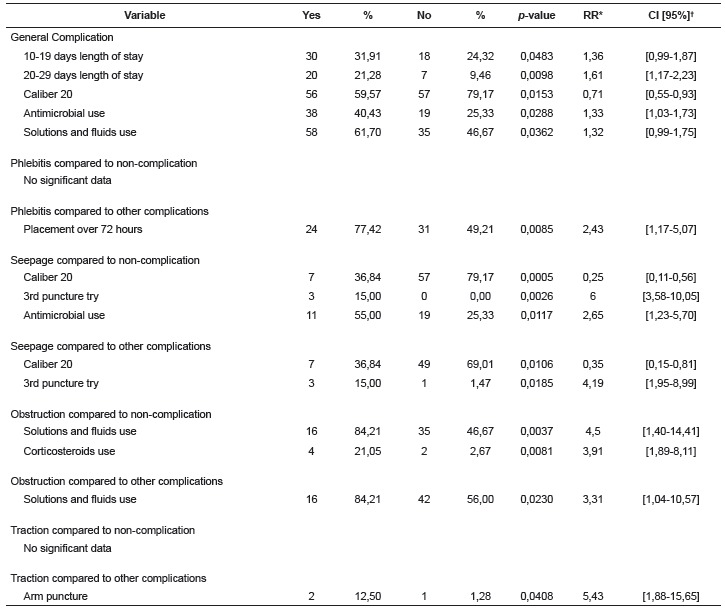
Description of risk factors statistically associated to the development of complications in both catheter groups. Curitiba, PR, Brazil, 2014

* RR Relative Risk; † CI Confidence Interval 95%

When crossing the data of all catheters that developed phlebitis with those who did not develop complications, no variable related to catheter data was statistically significant. In assessing the occurrence of phlebitis in total catheters, contrasting the data with catheters that developed other complications, the catheter placement period exceeding 72 hours was a risk factor for the development of phlebitis, increasing its risk by 2.43 (p = 0.0085) (Table 3).

By analyzing the catheters with seepage, compared to catheters that did not develop any complication, in total catheters, gauge 20 reduced the risk of seepage in 0.25 (p = 0.0005) compared to gauge 22. Catheters that had a successful puncture only after the third attempt increase the risk of seepage in 6 times, compared to a single attempt (p = 0.0026). The use of antimicrobials also ranked as a risk factor for this complication, increasing it by 2.65 (p = 0.0117). When comparing seepage with other complications in total catheters, significant risk factors showed to be: 20 Gauge reduces by 0.35 (p = 0.0106) compared to 22; and the third-try successful puncture increases by 4.19 (p = 0.0185) the risk of seepage (Table 3).

Comparing the obstructed catheters with those without any complications, putting together the total number of catheters, it appeared as the statistically proven risk factors for the appearance of this complication the use of infusion of solutions and fluids (RR = 4.5; p = 0.0037) and use of corticosteroids (RR = 3.91, p = 0.0081). Of the total surveyed catheters, catheters with obstruction versus other complications, had as risk factor for infusion of solutions and fluids (RR = 3.31; p = 0.0230). Correlating catheters with traction outcome with catheters that had no complications, on the total surveyed catheters, no risk factors were associated with the development of this complication. By comparing the catheters that developed traction with those who had other complications, on the total surveyed catheters, the region of the arm was shown as a risk factor compared to forearm (RR = 5.43; p = 0.0408) (table 3).

**Figure 1:**
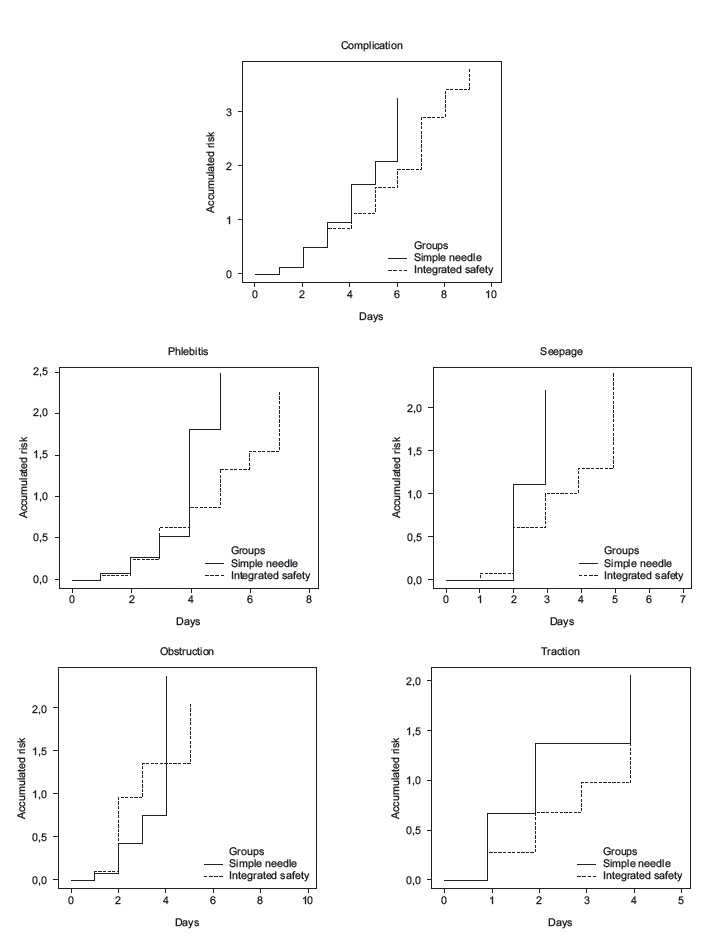
Cumulative risk curves for complications, phlebitis, seepage, obstruction and traction. Curitiba, PR, Brazil, 2014

Cumulative risk rates were estimated for all complications in the same manner for the four more frequent complications in this study. There was no significant difference between the curves (Figure 1). However, it was noted that after the third day, the risk in the group that used the integrated safety catheter is progressively shrinking, when compared to the group using simple needle catheter. There was similarity between the risks of phlebitis up to four days with the catheter, but from the fifth day on, phlebitis risk rate in the group with simple needle catheter was close to 2.0, while in the group with integrated safety catheter was 1.0. The cumulative risks rates for seepage are almost equal up to the second day, but after the third day the risk rate in the group with integrated security catheter was close to 0.5 while being 1.1 in the control group. In the case of obstructions, the cumulative risk rate is higher in integrated safety catheter group from the first day of puncture. Cumulative risk rates are lower related to develop traction in the integrated safety catheter group, and were were noticeable from the second day of placement of the catheter (Figure 1).

The data relating to complications were examined through multiple regression analysis using logistic regression. When referring to the total of catheters, there is a reduced risk for developing complications by 0.75 (p = 0.0350) for the gauge 20 catheter; an increased risk of 1.39 (p = 0.0351) for the infusion solutions and fluids; and increased risk of 1.40 (p = 0.0214) for the administration of corticosteroids. In analyzing the risk for complications, only in the group with integrated safety catheter, the variable diameter of the catheter decreased risk by 0.57 (p = 0.0007); the shift in which puncture was performed increased it by 1.51 (p = 0.0116); and the use of other drugs also increased it by 2.61 times risk (p = <0.0001). As for the group using simple needle catheter, the solutions and fluids administration variables and corticosteroids increased the risk of developing complications by 1.66 (p = 0.0298) and 3.08 (p = 0, 0130), respectively, while considering the variable use of other drugs, the risk was reduced by 0.66 (p = 0.0400) (Table 4).

**Table 4:** Poisson's regression with robust variance of complications in catheters' groups. Curitiba, PR, Brazil, 2014

Variable	Coef*	*p*-value	RR†	CI [95%] of RR^‡^
Total catheters				
Gauge 20	-0,29	0,0350	0,75	[0,57-0,98]
Use of solutions and fluids	0,33	0,0351	1,39	[1,02-1,90]
Use of corticosteroids	0,34	0,0214	1,40	[1,05-1,86]
Integrated safety catheter				
Gauge 20	-0,57	0,0007	0,57	[0,41-0,79]
Puncture in night shift	0,41	0,0116	1,51	[1,09-2,05]
Use of other drugs	0,96	<0,0001	2,61	[1,73-3,97]
Simple needle catheter				
Use of solutions and fluids	0,50	0,0298	1,66	[1,05-2,61]
Use of corticosteroids	1,12	0,0130	3,08	[1,27-7,47]
Use of other drugs	-0,41	0,0400	0,66	[0,45-0,98]

* Coef = Model coefficient; ^†^ RR = Relative Risk; ^‡^ CI = Confidence Interval.

## Discussion 

Regarding the socio-demographic profile, the population was homogeneous, predominantly Caucasian, age groups in the 50's, balance between the sexes, no smoking and alcohol abuse reports and absence of comorbidity, similar to the profile of several previous studies of patients evaluating peripheral venous catheters^3^ ^,^ ^9^ ^-^ ^15^. Regarding the characteristics of catheters, the studies indicate that the 20 Gauge is the most used^12^ ^-^ ^16^, located in the left upper limb^2^ ^,^ ^12^ and forearm region^1^ ^,^ ^10^ ^-^ ^11^ ^,^ ^15^, puncture success in the first attempt^1^ ^,^ ^3^, and permanence of the catheter over 72 hours^10^ ^-^ ^12^ ^,^ ^15^ ^,^ ^17^ similar to the findings of this research. This research was divergent with studies pointing to the frequent use of antimicrobials^1^ ^-^ ^3^ ^,^ ^16^ ^-^ ^17^. Similar rates of complications in the peripheral venous catheterization are found in 52%^9^ and 51.1%^3^ of catheters.

The findings show that the risk factors for the development of complications in relation to clinical variables were: length of hospital stay between 10-19 and 20-29 days. Data for this variable did not presented risk in other studies. When considering the caliber of the catheter inserted, the present research indicated that the 20G reduces the risk of complications compared to 22G. Other study corroborates the findings of this research stating that small caliber catheters (22G and 24G) are 1.84 times more likely to produce complications compared with large caliber (16G to 20G) (p < 0.0001)^18^. When performing multiple regression analysis in the total of catheters, we found decreased risk for the development of complications in the caliber of the catheter 20, fact that was repeated separately in the group with integrated safety catheter.

It should be noted that the use of antimicrobial agents increased the risk of complication in the catheter, just as infusion of solutions and fluids. In the multivariate analysis regarding the development of complications for total catheters, there was an increased risk for infusion solutions and fluids as well as for corticosteroids. Considering only the group that used the integrated safety catheter, the use of other drugs increased the occurrence of complications. In the group of simple needle catheter, the variables administration of solutions and fluids as well as corticosteroids increased the risk of complications, while the variable using other drugs, reduced this risk. Another study shows that medication infusion results in complications 1.41 times more frequent when compared to hydration solutions (p = 0.0006) both in univariate and multivariate analysis (RR = 1.60, p = 0.006)^18^. In the cumulative risk analysis it was perceived that the risk to develop complications in the integrated safety catheter group was increasingly smaller after the third day as compared to the simple needle catheter group.

By comparing the catheters that developed phlebitis with catheters developing other complications, having the catheter for more than 72 hours in place showed itself as a factor that increases risk. A study, linking the catheter time in the vein with phlebitis identified the development of this condition in 28% of catheters between the fourth and fifth days of stay (p = 0.03)^19^. Another study showed that the probability of phlebitis development increases by 5% every 24 hours the catheter remains inserted in the patient (15).

Related to the length of time passed with the catheter inserted, the multiple analysis of a study result in an Odds Ratio (OR) of 1.010 for periods longer than 72 hours (p < 0.001)^12^. Other authors say that a catheter inserted for less than 48 hours and by 49 to 96 hours has a positive risk of phlebitis of 5.8 (p = 0.000) and 2.8 (p = 0.002) respectively when compared to the period 97-120 hours^17^. A binary logistic regression showed an OR of 2.72 (p = 0.000) when the catheter is inserted for more than 48 hours^20^, contrasting with the previous study. The cumulative risk rate of this research showed doubled risk for the simple needle catheter group after the fifth day of the catheter insertion.

Divergent data to this research stress, through multiple analysis, that catheters placed in the antecubital fossa (OR = 0.66, p = 0.0260) and forearm (OR = 0.52, p = 0.0080) are less likely to develop phlebitis compared to those placed in the back of the hand^15^. Another study differs, referring that insertion in the forearm and arm compared to hand/wrist increases the risk of phlebitis by 1.53 (p = 0.024)^18^. A logistic regression analysis carried out in 2013 showed a reduction of the risk of phlebitis by 0.32 (p = 0.038) for catheters placed in upper limbs^21^. The stay of patients in orthopedic clinic (OR = 0.53, p = 0.034) and surgical wards (OR = 0.61, p = 0.041) reduce the risk of phlebitis when compared to those admitted to Medicine units^15^. Being woman is significantly associated with phlebitis through multiple analysis, thereby increasing the risk by a relative risk (RR) of 1.64 (p < 0.001)^1^, RR = 2.44 (p = 0, 0003)^18^, and RR = 1.9 (p = 0.007)^17^. The age decreased by 0.99 times the risk of phlebitis (p <0.001) ^1^. Presence of diabetes as comorbidity increased the chances of phlebitis (OR = 2.42; p = 0.011)^20^. A gauge 18G or wider catheter increased the risk of phlebitis by 1.48 (p = 0.014)^1^, as well as the presence of an infectious process in the patient (RR = 1.41, p = 0.022)^1^. The use of antimicrobial agents was also an increased risk factor for phlebitis by 1.48 (p < 0.01)^1^ 1.87 (p = 0.013)^21^ and 2.4 (p = 0.002)^17^. The infusion of other drugs reduced the risk of phlebitis in 0.79 times (p = 0.009)^1^, but comparing the infusion of drugs with hydration solutions, there is an increased risk of 1.55 (p = 0.02)^18^.

By analyzing the risk factors for the occurrence of seepage, comparing the catheters that showed seepage with those without complications, the third puncture try increased the risk by six times; the caliber 20G reduces the risk of seepage, while antimicrobial use increases it in the total of catheters. By comparing the catheters with seepage and catheters with other complications, there is a lesser risk for the use of 20G and an increased risk for the third try puncture. In survival analysis, after the third day of having the catheter placed, the cumulative risk of seepage was half for the integrated safety catheter group compared to the simple needle catheter group.

By comparing the catheters presenting obstruction with those catheters without any complication, infusing solutions and fluids, as well as corticosteroids increased the risk for this complication. Comparing the occurrence of obstruction with other complications for total catheters, infusing solutions and fluids increased the risk of obstruction. A study using multivariate analysis found significant relationships between the obstruction and gender=feminine (RR = 1.44, p < 0.001); puncture in the hand (RR = 1.47, p < 0.001); cubital fossa (RR = 1.27, p < 0.001) and arm (RR = 1.25, p = 0.016); antimicrobial use (RR = 1.41, p < 0.001), corticosteroids (RR = 1.36, p = 0.028) and antipyretics (RR = 0.76; p = 0.030) and infection of the patient (RR = 1.27, p < 0.001)^1^. When comparing the types of catheters, the cumulative risk rate was higher in the integrated safety catheter group from the first day of the catheter.

By comparing the catheters that had the outcome traction with those who did not present any complications, no significant data was obtained. When the traction was compared to other complications, the arm region was shown as a risk factor compared to the forearm. Other studies show risk factors different to those presented in this study, being related to the presence of two or more comorbidities (RR = 0.78, p < 0.05); 18G the bore (RR = 1.43, p < 0.01); puncture in the cubital fossa (RR = 1.99, p < 0.01) and hand (RR = 2.72, p < 0.01); antipyretic management (RR = 1.50, p < 0.05) and other drugs (RR = 1.26, p < 0.05)^1^. Multiple analysis of another study presented as risk factors the puncture site in the hand (RR = 2.45, p < 0.001) and cubital fossa (RR = 1.65, p = 0.001); 22G or the lower (RR = 1.29, p = 0.030)^1^. Regarding the traction survival analysis, in this research the cumulative risk rate is lower in the integrated safety catheter group since the second day on of the catheter.

The absence of records related to venipuncture in the patient charts limited this research. For this reason at the end of the pilot test and in addition to the readings in nursing notes, there was performed an active search for this information through a daily assessment in the wards of the surveyed units, just as questions directed to the officials and employees. Another limiting factor was the impossibility of blinding, due to the physical characteristics of catheters. The applicability of the research results is to assist the professional in choosing the best or most appropriate peripheral venous catheter technology, suitable to the prescribed patients therapeutics in the care process. The findings can permeate public policies, clinical guidelines, standards, protocols and procedures in patient care, in order to reduce the occurrence of complications.

## Conclusion 

Risk factors for the development of local complications in both catheter types, lengths of stay between 10 to 19 days, 20 to 29 days, and antimicrobial use of solutions and fluids. In the group that used the integrated safety catheter, hospital stays between 10-19 days and in the group with simple needle catheter, lengths of stay from 20 to 29 days and the use of solutions and fluids increased the risk towards the development of any complication. The cumulative risk rates are lower for the development of phlebitis, seepage and traction in the integrated safety catheter group, indicating the advantage of its use in infusing solutions and fluids, administration of corticosteroids and antimicrobials; no risk factors associated with obstruction was found. The simple needle catheter showed no risk factors related to the development of phlebitis, seepage and traction.

Therefore, it is recommended that the staff acquire specific training for insertion, maintenance and removal of catheters, in order to succeed in the first try and minimize the risk factors associated with complications; to puncture using preferably 20G caliber catheters in the forearm region, carry out careful monitoring of venous access in which are administered antimicrobial solutions and fluids, corticosteroids, vesicant drugs and electrolytes, and finally to properly record the complications of peripheral venous catheterization in a clear, objective and complete manner. These attitudes will maximize the survival of inserted catheters and decrease the adverse effects to the patient.
